# Exploration of the representation of the allied health professions in senior leadership positions in the UK National Health Service

**DOI:** 10.1136/leader-2023-000737

**Published:** 2023-08-24

**Authors:** Nicola Eddison, Aoife Healy, Nina Darke, Mary Jones, Millar Leask, Gwen L Roberts, Nachiappan Chockalingam

**Affiliations:** 1 Centre for Biomechanics and Rehabilitation Technologies, Staffordshire University, Stoke-on-Trent, UK; 2 Royal Wolverhampton NHS Trust, Wolverhampton, UK; 3 Yeovil District Hospital NHS Foundation Trust, Yeovil, UK; 4 Royal Berkshire Foundation Trust, Reading, UK; 5 Cardiff and Vale University Health Board, Cardiff, UK

**Keywords:** management, leadership assessment, role model

## Abstract

**Background:**

Allied health professionals (AHPs) are an important group within the National Health Service (NHS) in the UK and make up a large portion of the workforce. Investment in AHP leadership is believed to lead to improvements in patient care, resource use, collaboration and innovation. This study aims to assess the current state of AHP strategic leadership within the NHS.

**Methods:**

A freedom of information (FOI) request was sent to all NHS Trusts and health boards (HBs) within the UK NHS. The questions focused on the AHP workforce, with a particular interest in the chief AHPs (or equivalent roles) working in an NHS setting. Analysis of the FOI used a range of descriptive statistics.

**Results:**

Of the 217 Trusts/HBs contacted, responses were received from 160 (74%). The majority (81%) reported that they employed a Chief AHP or equivalent role, with only 14% of these having a position on the Trust/HB executive board. There were 50 different job titles reported as the titles for the chief AHP or equivalent roles: with director of AHPs (18.6%), lead AHP (13.9%) and chief AHP (11.6%) being the most reported titles. The results identified an inequity of representation of AHP professions within senior AHP leadership; with most of these roles (70%) held by physiotherapists and occupational therapists.

**Conclusion:**

Changes in AHP strategic leadership are needed to address the inequities identified in this study. Addressing these issues is required to enable inclusive leadership, which is crucial to improve the contribution of AHPs to healthcare.

**WHAT IS ALREADY KNOWN ON THIS TOPIC**

- Allied health professionals (AHPs) are a group of distinct and diverse healthcare professions and together form the third biggest workforce in the UK National Health Service (NHS). Despite this, strategic leadership positions for AHPs within the NHS have been relatively small. From a systems perspective, understanding the current state of AHP strategic leadership within the NHS and the barriers to training and success will help.

**WHAT THIS STUDY ADDS**

- This study provides comprehensive data on how many NHS Trusts/health boards (HBs) have appointed a chief AHP (or an equivalent role), and whether that role has a position on the Trust’s/HB’s executive board. Also, the report highlights, the diversity of professions represented across this role.

**HOW THIS STUDY MIGHT AFFECT RESEARCH, PRACTICE OR POLICY**

- Changes in AHP strategic leadership are needed to address the inequity in the AHP professions represented in the chief AHP role, the non-standardisation of a title for chief AHPs and the lack of representation of chief AHPs on Trust/HBs executive board identified in this study. Addressing these issues is required to enable inclusive leadership, which is crucial to improve the contribution of AHPs to healthcare.

## Background

 In recent years, the National Health Service (NHS) has increasingly focused on the importance of allied health professionals (AHPs) in transforming healthcare,^1 2^ partly driven by the changing pressures on the healthcare service such as increased life expectancy, alongside increasing comorbidities such as obesity^3^ and diabetes.^4^ AHPs are a group of distinct and diverse healthcare professions, totalling 185 000 professionals currently working within the NHS.^5^ In England, there are 14 allied health professions (art therapists, drama therapists, music therapists, chiropodists/podiatrists, dietitians, occupational therapists, operating department practitioners (ODPs), orthoptists, osteopaths, paramedics, physiotherapists, prosthetists and orthotists, radiographers and speech and language therapists).^6^ This varies in Wales as they include practitioner psychologists but not ODPs, osteopaths or radiographers and categorise prosthetists and orthotists separately.^7^ In Northern Ireland and Scotland, prosthetists and orthotists are also categorised separately and ODPs and osteopaths are not included.^8 9^ The professions vary in workforce sizes, the largest workforce is physiotherapists, at 61 132, with the smallest workforces, orthoptists and prosthetists/orthotists, numbering between 1000 and 2000.^10^ In comparison, there are 704 520 registered nurses.^11^

Although AHPs are the third largest workforce in the NHS,^12^ traditionally strategic leadership positions for AHPs within the NHS have been relatively small in number.^13^ The majority of NHS leadership positions have historically been filled by medical professionals.^14^ As healthcare providers adapt to the changing health requirements of the nation, AHPs have been identified as ideally placed to meet these needs and drive change.^2 15^ Since 2017, NHS England has published documents promoting the benefits of professional diversity at the executive board level and the recommendation of a chief AHP role in each Trust to help use the potential of this workforce.^12 14^

The lack of leadership within the allied health professions is not specific to the UK and current literature,^16^,^20^ is unequivocal in identifying this issue. Previous reports focus on local leadership,^21^ leadership in multidisciplinary teams and leadership education.^22^ Information is lacking on AHP strategic leadership at the executive board level, such as Chief AHPs. There are a multitude of reasons why strategic leadership for AHPs is essential. Clinical leaders in healthcare are at the forefront of decision-making and have significant responsibilities such as organisational planning^15^ and deciding how limited funding is spent.^17 23^ They ensure that an organisation is ready to meet future healthcare challenges^24^ and have the power to drive change and facilitate the work of their colleagues. Leadership from those who understand the capabilities of the workforce they represent are essential to influence decision-making within strategic leadership.^24^

Furthermore, effective strategic leadership has been shown to improve performance and quality of care and promote efficient use of resources.^25 26^ It also ensures that represented professions can compete for the resources they require.^15^ Competent leadership may also improve AHP visibility, influence, profile, and status,^27^ improving job satisfaction, engagement, and motivation^26 28^ and subsequently improving staff retention rates.^15^ Therefore, representation within strategic leadership roles is key in demonstrating the value of AHPs.^29^

In England, NHS Trust executive boards are required to include a medical and a nurse director, but there is no obligation to include clinicians from other professions. As a result, board-level accountability for AHPs is the responsibility of the director of nursing or chief nurse.^20^ This differs in Wales where, since 2009, boards must include an officer responsible for therapies and health sciences.^30^ The lack of diversity within senior leadership roles hinders the ability of AHPs to develop and demonstrate the skills required to become leaders at the executive board level.^2 31^ In turn, this shortage of AHP leaders in strategic roles creates a lack of role models and mentors to inspire and give AHP clinicians the confidence to pursue leadership opportunities.

When AHP clinical leaders are integrated into operational governance systems, they are influential in supporting models of care that improve outcomes for patients.^15^ In contrast, when allied health leaders were not included in planning, some patients who would have benefited from allied health intervention were not offered access to the service.^15^ Where Trusts have appointed a chief AHP, there is significant evidence that the ‘value and contribution of the workforce is immediate’^28^; including, unlocking the potential of AHPs within the Trust and influencing the AHP workforce, and increasing its engagement in improving services.^13^ The Allied Health Professions Strategy for England^1^ states that evidence now demonstrates that having a chief AHP role in an organisation is crucial to the delivery of quality care. Similarly, the literature shows where there is a lack of AHP strategic leadership there is reduced visibility and utilisation of this workforce, including patients not benefiting from AHP services, and preventing opportunities to identify benefits from these professions’ skills and expertise.^15^

Allied health professions into action^12^ encouraged AHPs to fill formal leadership positions to deliver change and produce a more balanced representation within management structures. The AHPs strategy for England^1^ stated that diverse board and senior teams increase the likelihood that the ambitions of the NHS Long Term Plan are realised and that AHPs needed to have diverse leadership and opportunities to become leaders. However, one should note that there are variations in the starting grades and the progression through their careers across all 14 allied health professions. This could contribute to difficulties in eligibility to apply to senior leadership roles. There has been a plethora of research to explore the links between leadership and identity focusing on belongingness and the value of diversity^32^ which leads to psychological empowerment and work group identification. Further work highlights the importance of leader inclusion for employees who are members of marginalised social identity groups.^33^

This study aims to determine the current state of NHS AHP strategic leadership including how many NHS trusts/health boards (HBs) have appointed a chief AHP (or an equivalent role), and whether that role has a position on the Trust’s/HB’s executive board. This study also investigates the diversity of professions represented across this role. The exploration of professional diversity in AHP strategic leadership roles aligns with the recently published NHS England AHP strategy.^1^ It is important to ensure AHP professions are not inadvertently overlooked, by a lack of representation, to ensure all the constituent professions have a voice and influence. There is currently no available data on the professional background of those who currently occupy chief AHP (or equivalent) roles in the NHS. It is important to capture this data to understand whether there is a disparity of representation and whom this might impact.

## Methods

In March 2022, a freedom of information (FOI) request^34^ was sent to the 217 Trusts and HBs within the NHS in the UK.^35^ Due to the nature of this work, there was no patient and public involvement.

The FOI consisted of 12 questions in total, 9 of which were closed-ended questions and 4 were open-ended questions (see online supplemental material). The survey focused on the AHP workforce, with a particular interest in the chief AHPs (or equivalent roles) working in an NHS setting. The questions focused on the following areas: (1) if the Trust or HB had a role which resembled the Chief AHP description provided, (2) the job title of the role, (3) the professional background of the post holder (if applicable), (4) if the position was included on the Trust board, (5) which of the allied health professions the Trust/HB employed and (6) If the Trust/HB does not have a chief AHP role (or equivalent) the reason(s) for this. While the data itself do not lend itself to formal statistical analysis, we have used a range of descriptive statistics such as percentages and range to showcase the data.

## Results

**Figure 1:**
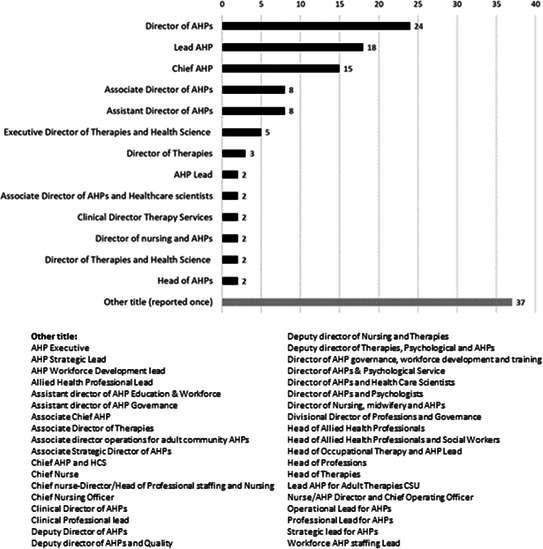
Variation in job titles for roles matching the description of "chief AHP". AHP, allied health professional; CSU, Clinical Service Unit; HCS, Health Care Science.

Of the 217 Trusts/HBs contacted, 160 responses were received, a 74% response rate. Most responses did not provide an answer for Q 3.3 which asked for an opinion and is therefore beyond the remit of an FOI request, consequently, this question was excluded from the analysis. The majority of the Trusts/HBs included in the analysis (130/160; 81%), reported that they have a chief AHP role or equivalent which was comparable to the job summary included in the FOI request. Nearly one-fifth of Trusts/HBs (30/160) reported that they did not have a comparable role. Regarding job titles pertaining to the role of ‘chief AHP’ (or equivalent), 50 different titles were reported across the 130 Trusts/HBs reporting to have such a position. The most common titles were director of AHPs (24/130; 18%), lead AHP (18/130; 14%) and chief AHP (15/130; 12%), these titles accounted for 44% (57/130) of all responses (see figure 1).

**Figure 2:**
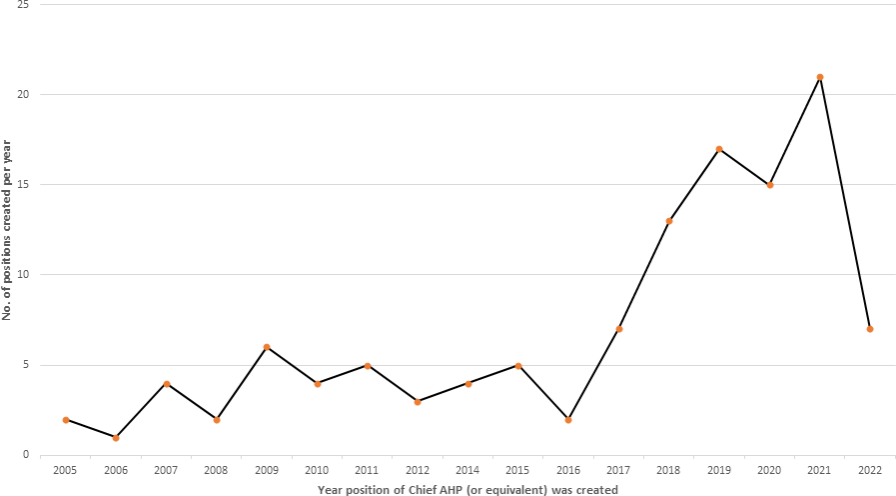
Breakdown of the year each position matching the ‘chief AHP’ role description was created. AHP, allied health professional.

The results showed that the first job roles matching the description of a chief AHP (or equivalent) role were created in 2005 (2/130; 1.5%), with 67% (87/130) of roles created in the last 8-year period (see figure 2). In 2021, 16% (21/130) of chief AHP (or equivalent) roles were created, which constitutes the largest growth in a single year. In the NHS across the UK, of the Trusts/HBs with chief AHP role (or equivalent) roles who responded to this study, 96% (125/130) reported that the role was currently occupied.

**Figure 3:**
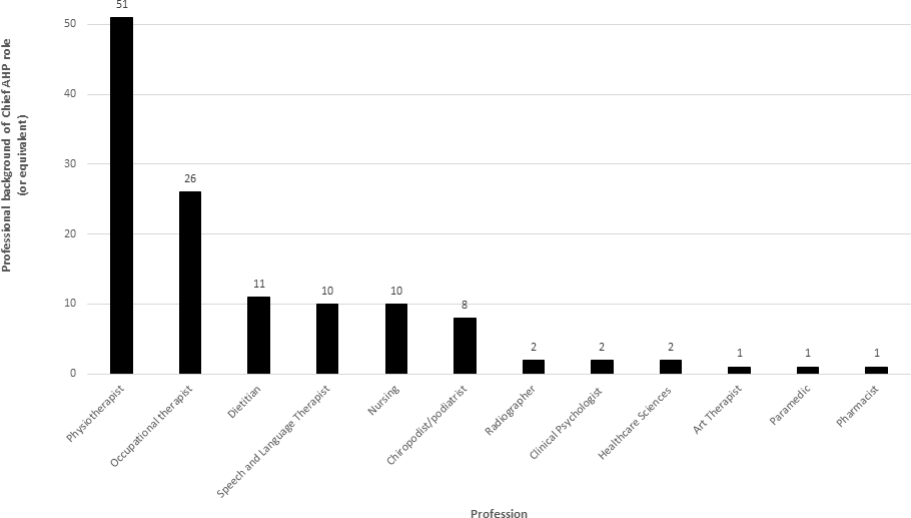
Professional background of chief AHP role (or equivalent). AHP, allied health professional.

Most of the people (110/130; 85%) who currently occupy these roles were registered AHPs, with the majority being physiotherapists (51/110; 46%), followed by occupational therapists (26/110; 23.6%). Thus, 70% (77/110) of the AHP registered staff occupying a chief AHP role (or equivalent) as reported by the Trusts/HBs in this study, are physiotherapists and occupational therapists. While drama therapists, music therapists, operating department practitioners, orthotists/prosthetists, orthoptists and osteopaths do not hold any of the 110 AHP occupied chief AHP (or equivalent) posts in the NHS in the UK, included in this study. Of the remaining 13% of occupied posts, the post holders are registered nurses, clinical psychologists, health scientists, healthcare sciences and pharmacists, with registered nurses accounting for the largest proportion at 8% (10/130) (see figure 3).

**Figure 4:**
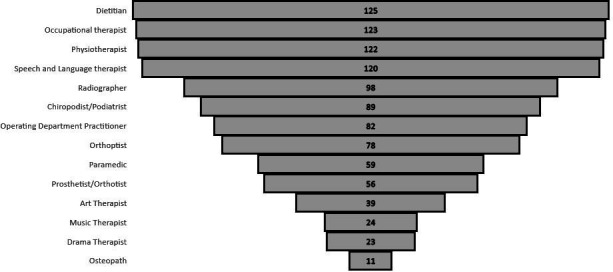
AHP professions employed by each Trust/HB. AHP, allied health professional; HB, health board.

Only 17% (22/130) of the Trusts/HBs who responded to the FOI employed a minimum of 10 of the AHP professions and only one employed all 14 professions. While 12% (16/130) of Trusts/HBs employed at least five AHP professions with a mean average of 7.9 different AHP professions being employed across the UK NHS Trusts/HBs. Dietitians (96% 125/130), occupational therapists (95%; 123/130), physiotherapists (94%; 122/129) and speech and language therapists (92%; 120/130) were the most employed AHP professions across the UK. While paramedics (45%; 59/130), prosthetists/orthotists (43%; 56/130), art therapists (30%; 39/130), music therapists (18%; 24/130), drama therapists (18%; 23/130) and osteopaths (8%; 11/130) are employed by less than 50% of NHS Trusts/HBs in the UK (see figure 4).

Of the 24 Trusts/HBs employing 10 or more of the AHP professions, physiotherapy and occupational therapy accounted for 54.2% (13/24) of the AHP disciplines occupying the chief AHP role (or equivalent) at these Trusts/HBs. A minority of the roles (3/24; 12.5%) are occupied by staff from a non-AHP background. Of those Trusts/HBs employing five or fewer of the allied health professions (15/129), 60% (9/15) of the chief AHP roles (or equivalent) are occupied by physiotherapists and occupational therapists. While 20% (3/15) of the roles are occupied by staff from a non-AHP background.

Within the Trusts/HBs who reported having a chief AHP role (or equivalent), 86% (112/130) did not have a position on the Trust board. Of the 17 chief AHP roles (or equivalent) with a position on the Trust board, 8 were occupied by a staff member with a non-AHP background; 7 had a nursing background and 1 with a health scientist background. Of the nine roles occupied by a staff member with an AHP background six were from Welsh HBs.

Finally, of the 30 Trusts/HBs that reported that they do not have a current job role equivalent to a chief AHP role, 24 (80%) did not envisage advertising or creating a similar job role within the next 6 months. Although 19 of the 24 Trusts/HBs (79.2%) reported they are aware of the NHS England and NHS Improvement (NHSI) strategy to have designated senior AHP leads.^36^

## Discussion

An ‘enhanced foundation’ of the 2022 AHP Strategy for England^5^ is the promotion of diverse and inclusive leadership of AHPs within executive boards and senior teams, along with the removal of practices that ‘prevent AHPs from harnessing their leadership ability’. The importance of effective clinical leadership cannot be underestimated. A lack of effective clinical leadership has been seen as a contributing factor in incidents of poor-quality care and adverse outcomes.^37 38^ While investment in leadership is considered to promote innovation, collaboration, improved care and better use of resources.^19^

In 2018, NHS England recommended that strategic AHP leaders were appointed within provider organisations,^1^ and more recently, within integrated care systems.^39^ The aim is to provide the AHP workforce with greater visibility and voice and to ensure that the workforce can contribute to system-wide policies and initiatives.^39^ However, the diversity of the allied health professions means that each group might need its own representatives to ensure the priorities and needs of each group are heard, and their individual contributions to Trust/HBs priorities are not overlooked.

The results of this study confirm that there is an inequity of representation of the allied health professions within senior AHP leadership. It was found that physiotherapists (who account for 25% of registered AHPs^40^) occupy 46% of the Chief AHP roles occupied by AHPs and occupational therapists (who account for 17% of registered AHPs^40^) occupy 24%. Therefore, with 70% of the AHPs in chief AHP (or equivalent) roles being from either a physiotherapy or occupational therapy background, questions must be asked regarding why clinicians from other allied health professions are not being represented within these leadership positions.

Further, seven of the allied health professions were not represented at all, our results show that the professions with the smallest workforces (those with under 5000 registrants) are not currently represented. These include drama therapists, music therapists, prosthetists and orthotists, orthoptists and osteopaths.^40^ However, it is not quite as straightforward as just the size of the workforce being the reason for the lack of representation. For example, the AHP profession with the third largest workforce is radiography with a very similar number of registrants compared with occupational therapy. However, just two chief AHP roles are currently held by radiographers while 26 are held by occupational therapists. Conversely, dietitians have a workforce of just over 10 000 registrants but currently hold 10 chief AHP roles while operating department practitioners have a workforce of 15 000 but are not represented within chief AHP roles.

The results from this study show that not all AHP professions were employed by all the Trusts/HBs who responded. We found that dietitians, occupational therapists, physiotherapists and speech and language therapists were employed by the most Trusts/HBs and it is these four professions which were also found to occupy the four highest numbers of chief AHP roles. Likewise, prosthetists and orthotists, music therapists, drama therapists and osteopaths were among those employed by the least numbers of Trusts/HBs. In the instances where the chief AHP role has been filled by a member of a profession with a small workforce that is, art therapist, three of the four Trusts/HBs who responded were specialist Trusts/HBs (ie, they specialise in a specific condition such as cancer or neurology). Anecdotally, based on the authors’ experiences, in some Trusts/HBs, due to workforce shortages, sometimes AHPs are used to cover service provision typically provided by a different allied health profession (eg, a specialist podiatrist prescribing ankle foot orthoses which would typically be provided by an orthotist). While there are instances where this might be acceptable, further forward referrals should be organised either to multidisciplinary teams or specialist services for the benefit of the patient. This seldom happens and appropriate AHP leadership would have the potential to change this dynamic.

The combination of being both part of a smaller workforce and not being employed by the majority of Trusts/HBs may mean there is less familiarity with and understanding of these allied health professions among senior management, other clinical teams within the Trust/HB, and patients. Potentially resulting in those professions being less well integrated within the hospital structures, creating a potential barrier to those in the professions with smaller workforces reaching senior leadership roles. This is exacerbated in the case of some professions (such as prosthetists and orthotists) which are often contracted services meaning that their workers are not direct employees of the Trust/HB.^41^ There is also the potential that unfamiliarity is a reason that these smaller workforces are employed by fewer Trusts/HBs. In a survey investigating occupational therapists’ contributions to palliative care, it was noted that when colleagues had an increased understanding of what an occupational therapist can contribute, it led to occupational therapists expanding their scope of practice.^42^ Raising the profile of AHP skillsets may lead to their specialisms being used in a wider scope of healthcare environments.

The presence of AHPs within senior leadership roles increases the visibility of the AHP professions and provides credibility with Trust/HB boards, as well as demonstrating that those professions are valued^43^ and considered able to contribute to service redesign and improvements. The results of this study found that 19% of the Trusts/HBs who responded do not currently have a Chief AHP or equivalent role, and 80% of those did not envisage creating one within the next 6 months. Regarding job titles for AHP leads, in 2018 it was reported that there were 41 job titles across 43 job descriptions for the most senior AHP lead^44^; meaning that largely no ‘readily identifiable ‘go-to’ AHP leader’ existed within the NHS, as there is for other professions such as nursing. The current study identified 50 different job titles for equivalents of chief AHP roles, indicating that standardisation of this title is still an issue.

A lack of visible role models within senior leadership contributes to a reduced sense of identity and leaves AHPs without a readily identifiable leader as exists for other professions (eg, the director of nursing).^43 45^ The absence of professions with smaller workforces in these roles creates a continuing problem with culture and a perception that leadership opportunities are not open to them^45 46^ which in turn contributes to the absence itself. Leader inclusion is shown to be effective in facilitating an inclusive work environment, increasing positive employee attitudes and enhancing performance.^33^

A further complication is the lack of clarity regarding which professions are classified as AHPs. There are significant differences between the four nations of the UK, with some professions only having been recently added to the list. Even within England, some Trusts will include more than the 14 professions recognised by NHS England,^6^ such as psychologists, psychotherapists, biomedical scientists and social workers.^44^ This is also true internationally, with the USA and Australia including a wider range of professions.^47^ Furthermore, the term ‘therapies’ is often used as a synonym for AHPs which creates yet more confusion.^48 49^ As there is no universally accepted definition for the allied health professions, this impacts further on the sense of identity of AHPs, in particular, those from the professions with smaller workforces, and also the understanding of their roles and the contribution they can make within healthcare systems.

Another possible barrier for the smaller AHP professions being appointed to senior leadership positions is that those in these professions are less likely to have received leadership training during their career than the AHP professions with larger workforces, who, in turn, are less likely to have received training than nurses or medics.^45^ This is partly because as a collection of diverse professions, AHPs work in a variety of different settings and have less defined career pathways when compared with medical and nursing professions.^14 46^ A degree of serendipity has been reported in the career paths of those who have reached senior leadership positions, with knowledge having been acquired in ‘unstructured opportunities’^45^ due to a lack of developmental leadership roles being available for AHPs.^14^

As a result, AHPs (particularly those from professions with smaller workforces) can feel inadequately prepared or qualified for leadership positions^38 50^ with a lack of a clear structure to support leadership development.^51^ With the different career structures come significant variations in opportunities to develop leadership and management skills.^31^ For example, AHPs are usually employed in direct patient care roles, with fewer opportunities to progress into non-clinical areas.^38^ While in the current study occupational therapists occupied nearly a quarter of the chief AHP (or equivalent) roles, Eva and Morgan^42^ found occupational therapists across Europe spent their time on direct and indirect patient care activities, with limited ability to participate in leadership activities, and this will be true for other AHP groups. Traditional AHP career pathways tend to focus on the development of clinical skills, research or specialisation which may ‘reduce opportunities for operational and strategic leadership’.^14^

AHPs can find fewer career progression opportunities open to them; a recent NHS report noted that access to developmental leadership roles tends to be restricted to nursing or medical professions.^14^ Further, some posts are open to applicants from certain professions only, either excluding some AHP groups or not being open to AHPs at all.^28 44^ When chief AHP posts are open to AHPs, some Trusts have reported issues with not being able to appoint candidates due to gaps in their skills, knowledge and experience.^14^ Wylie and Gallagher^52^ found that AHPs who had received leadership training exhibited more transformational behaviours than those who had received none, meaning that they are more likely to possess the skills needed to contribute to healthcare planning and service delivery. Overall, it is recognised that leadership development within the NHS is ‘uncoordinated and inconsistent’.^45^ For AHPs, it has been recommended that leadership roles should be promoted as part of (rather than alternatives to) clinical progression and that training opportunities should be available for AHPs earlier in their careers, even in preregistration education.^14 22^ This would provide reassurance to AHPs that moving into leadership positions would not be a threat to their values or clinical credibility^36 46^ as these moves would be established and normalised steps in their career pathways, just as they now are for professions such as nursing. The NHS now provides opportunities open to AHPs to develop their leadership skills.^53 54^

Results found that 86% of those in Chief AHP (or equivalent) roles did not have a position on the Trust/HBs executive board. Of the nine chief AHPs (or equivalent) roles who had both an AHP background and a position on the Trust executive board, six were from Welsh HBs, which is due to the requirement in Wales to include an officer responsible for therapies and health sciences on executive boards.^30^ This shows that a change in NHS policy regarding which positions must be included on the board does have a positive impact on the inclusion of AHP leadership positions. Reducing constraints on AHPs to develop and demonstrate their senior leadership potential.^31^

As there are few chief AHPs (or equivalent) roles with a place on a Trust/HB executive board, responsibility for AHPs generally lies with the director of nursing or chief nurse.^20^ However, those from a nursing background may be unable to adequately represent AHPs at the executive board level, partly due to the extent of the nursing agenda,^44^ and partly because they are unlikely to have an in-depth understanding of the scope of practice for each of the of professions classified as AHPs. This is also due to the diversity across various AHPs and the marked differences between them. The chief AHP role can provide the necessary knowledge, reduce the fragmentation, and bring the workforce together,^28^ but this would be even more effective if all the Chief AHPs had a place at the executive board level.

NHS England has raised the profile of AHPs in recent years, ensuring that the term ‘AHP’ has a position and purpose within the clinical workforce of the NHS. However, as all the allied health professions are not represented in the most senior roles, it introduces the risk that the term ‘AHP’ will become synonymous with the professions with the largest workforces.

## Conclusion

This study is the first to map the state of NHS AHP strategic leadership in the UK; identifying inequity in the AHP professions represented in this role, non-standardisation of a title for chief AHPs and a lack of representation of Chief AHPs on Trust/HBs executive board. Changes are needed to enable AHPs to achieve ‘impactful, inclusive leadership’ which is identified as crucial to improve the contribution of AHPs to healthcare.^1^

### Recommendations

- To establish AHP leaders within Trusts/HBs, a standardised job title for the person leading AHPs within a Trust/HB (i.e., chief AHP) is required.
- A change in policy to stipulate a place for the chief AHP on Trust/HB executive boards, as currently exists in Wales, is required. This would bring AHPs in line with nurses and medical practitioners.
- An exploration of the barriers preventing all AHP professions from being represented in the chief AHP role is needed. With the aim of ensuring a wider representation of the AHP professions in chief AHP roles.
- The most senior AHP positions should be open to all the allied health professions, as should training and development opportunities, and early career pathways.

## Supplementary material

10.1136/leader-2023-000737 online supplemental material 1

## Data Availability

Data are available on reasonable request.
