# Supplementary material for: Exploration of the representation of the allied health professions in senior leadership positions in the UK National Health Service
Source: BMJ Lead. 2023 Aug 24;8(2):e000737. doi: 10.1136/leader-2023-000737 (PMC12038103; doi:10.1136/leader-2023-000737)
Supplement: online supplemental material 1 [file leader-8-2-s001.pdf]

The role of Director/Chief/Lead of AHPs is commonly described as *“Providing Workforce Leadership for Allied Health Professions, map the current AHP workforce across the trust, work with other trust AHP Directors and develop a strategic workforce plan for recruitment and retention of Allied Health Professions within the trust.” (1)*

Section 1. Within your Trust, do you have a role that meets the above description?

☐ Yes
☐ No

If the answer is “Yes” go to Section 2 and if the answer is “No” go to Section 3 only.

Section 2

2.1. What is title?

☐ Director of AHPs
☐ Chief AHP
☐ Lead AHP
☐ Other (please specify): ____

2.2 When was the role of Director/Chief/Lead of AHPs or its equivalent first created within the Trust?

____

2.3. Is there someone currently in this post?

☐ Yes
☐ No

If no, why not?

____
____
____
____
____
____
____

2.4. If yes, are they registered as an allied health professional (AHP)?

☐ Yes
☐ No

2.5 If yes, which profession?

☐ Art Therapist
☐ Drama therapist
☐ Music therapist
☐ Chiropodist/podiatrist
☐ Dietitian
☐ Occupational therapist
☐ Operating Department Practitioner
☐ Orthoptist
☐ Osteopath
☐ Paramedic
☐ Physiotherapist
☐ Prosthetist
☐ Orthotist
☐ Radiographer
☐ Speech and language therapist

2.6. If they are not registered as an AHP, what is their professional background?

2.7. Does this individual have a position on the Trust board?

☐ Yes
☐ No

2.8. Which Allied Healthcare professions are employed by your Trust?
(Please complete the table below)

	Select the AHP professions employed by your Trust	If your Trust employs this profession, select if the Director/Chief/Lead of AHPs or its equivalent leads/directs this profession
Art Therapists	☐	☐
Drama therapists	☐	☐
Music therapists	☐	☐
Chiropodists/podiatrists	☐	☐
Dietitians<br>Occupational therapists	☐	☐
Operating Department Practitioners	☐	☐
Orthoptists	☐	☐
Osteopaths	☐	☐
Paramedics	☐	☐
Physiotherapists	☐	☐
Prosthetists	☐	☐
Orthotists	☐	☐
Radiographers	☐	☐
Speech and language therapists	☐	☐

Section 3.

It has been shown that “there are benefits to improvement activity, as well as to the visibility and influence of the AHP workforce on the Trust’s priorities when there is a designated AHP lead”, and that these roles should be put in place in each Trust[1,2].

3.1. Do you expect to advertise/create a job role meeting the above description within the next 6 months?

☐ Yes
☐ No

3.2. If no, are you planning to advertise/create this role in the long term 12-24 months?

☐ Yes
☐ No

3.3 If no, what has been the main barrier/s that are preventing a job role meeting the above description from being created in the long term (12-24months)?

3.4. Is your Trust aware of the NHS England and NHS Improvement strategy (2019 Investing in chief allied health professionals: insights from trust executives.) to have designated AHP leads?

☐ Yes
☐ No

References:

1. Palma S, Harding D, Treadwell L. Investing in chief allied health professionals: insights from trust executives: a guide to reviewing AHP leadership for trust boards and clinicians.
2. Palma S, Harding D, Treadwell L. Developing allied health professional leaders: a guide for trust boards and clinicians.
